# The relation of sexual function to migraine-related disability, depression and anxiety in patients with migraine

**DOI:** 10.1186/1129-2377-15-32

**Published:** 2014-05-27

**Authors:** Defne Eraslan, Pınar Yalınay Dikmen, Elif Ilgaz Aydınlar, Cem İncesu

**Affiliations:** 1 Istanbul Psychiatry Institute, Istanbul, Turkey; 2 Department of Neurology, Acıbadem University School of Medicine, Acıbadem Maslak Hospital, Büyükdere Caddesi. No:40. Maslak/Sarıyer, 34457 Istanbul, Turkey; 3 Department of Psychiatry, Acıbadem University, School of Medicine, Istanbul 34457, Turkey

**Keywords:** Migraine, Sexual dysfunction, Migraine related disability, Depression, Anxiety

## Abstract

**Background:**

Depression and anxiety are two phenomena that affect quality of life as well as sexual function. Depression and anxiety levels are reported to be high in migraine sufferers. We aimed to understand whether sexual function in women with migraine was associated to migraine-related disability and frequency of migraine attacks, and whether this relationship was modulated by depressive and anxiety symptoms.

**Methods:**

As migraine is more commonly seen in females, a total of 50 women with migraine were included. The diagnosis of migraine with or without aura was confirmed by two specialists in Neurology, according to the second edition of International Headache Society (IHS) International Classification of Headache Disorders (ICHD-II) in 2004. Migraine disability assessment scale score, female sexual function index scores, Beck depression inventory score and Beck anxiety inventory scores.

**Results:**

Mean MIDAS score was 19.3 ± 12.8, and mean number of migraine attacks per month were 4.3 ± 2.7. Mean Female Sexual Function Index score was 20.9 ± 5.9 and 90% of patients had sexual dysfunction. Sexual dysfunction was not related to MIDAS score or frequency and severity of attacks. No relationship between sexual function and anxiety was found, whereas severity of depressive symptoms was closely related to sexual function. Depressive symptoms affected all dimensions of sexual function, except for pain.

**Conclusion:**

Sexual dysfunction seemed to be very common in our patients with migraine, while not related to migraine related disability, frequency of attacks and migraine severity or anxiety. The most important factor that predicted sexual function was depression, which was also independent of disease severity and migraine related disability. While future larger scale studies are needed to clarify the exact relationship, depressive and sexual problems should be properly addressed in all patients with migraine, regardless of disease severity or disability.

## Background

Given that chronic illnesses are related to sexual dysfunction [1], migraine as a chronic condition mainly seen in women is known to negatively affect quality of life [2] as well as sexual function [3-6]. Several studies have investigated the relationship between headache and sexual function. In one study, university students with migraine had reported more frequent sexual intercourse and reduced sexual satisfaction [3]. In another study, it was found that patients with either migraine or tension type headache experienced a considerable decline in multiple areas of sexuality [4]. This finding was supported in another clinical sample where women with both types of headaches reported high rate**s** of sexual symptoms and sexual distress [5]. This study also showed that both patient groups with tension type headache and migraine had high levels of anxiety and depression, but that depression did not show a positive correlation with sexual distress in migraineurs. On the other hand, some authors have suggested that migraine sufferers have higher levels of sexual desire than those suffering from tension type headache [6].

Depression and anxiety are two phenomena that affect both overall and sexual functions [7,8]. Depression and anxiety levels were reported to be high in migraine sufferers [9] while migraine has been indicated to cause disability and affect functioning in many areas of life [10].

However, is has not yet been exactly clarified how the sexual problems reported in patients with migraine are modulated: Are they the result of depression and anxiety that so often accompany this disorder; a natural consequence of the disability that severe forms of migraine creates in many aspects of daily life; or the result of a common pathophysiological background that underlies both migraine and sexual dysfunction?

To better understand this relationship, we aimed to find out whether sexual function in women with migraine was associated to migraine-related disability and frequency of migraine attacks, and whether this relationship was modulated by depressive and anxiety symptoms.

## Method

A total of 50 women were referred to the headache center of the Department of Neurology, Acıbadem University School of Medicine between May 2011 and May 2012 and fulfilled the patient selection criteria to be included in this non-interventional cross-sectional study. We selected female patients only, because migraine is mainly seen in women and most of our patients in the outpatient department are female. The diagnosis of migraine with or without aura was confirmed by two specialists in Neurology, according to the second edition of International Headache Society (IHS) International Classification of Headache Disorders (ICHD-II) in 2004 [11]. In order to be eligible for the study, the patients had to be between 18 and 50 years of age, with a history of migraine for at least six months and in a sexual relationship for the last six months. We excluded patients with tension type headache, diabetes mellitus or hypertension or any other major medical condition that is unstable and may affect sexual function, patients who were pregnant, breastfeeding or postmenopausal. In order to exclude any patient who were using antidepressants or any other medication known to have an effect on sexual function within the last 1 month, we asked for a complete list of medications used by the patient and checked each medication for possible sexual effects. The agents that caused an exclusion of a patient from the study included, but were not limited to typical antipsychotics, antimanic agents such as lithium carbonate, some antihypertensive medications and antiepileptics. We asked the patients whether they had been previously diagnosed with depression and excluded those who reported previous depression themselves or whose medical reports showed that they had a previous diagnosis of depression. Medication overuse headache was also excluded.

Written informed consent with an emphasis on anonymity of survey and interview results was obtained from each subject following a detailed explanation of the objectives and protocol of the study, which was conducted in accordance with the ethical principles stated in the “Declaration of Helsinki” and approved by the institutional ethics committee of Acıbadem University.

### Main outcome measures

After checking their eligibility, patients who gave informed consent were interviewed by clinicians to fill out the sociodemographic data form. The participants were then escorted to a private room to complete the self report study materials including questionnaires composed of sociodemographic data form, migraine disability assessment scale (MIDAS) [12,13], female sexual function index (FSFI) [14,15], Beck depression inventory (BDI) [16,17] and Beck anxiety inventory (BAI) [18,19] forms. We preferred to use self report instruments for sexual dysfunction, depression and anxiety, because we believed that it would result in more women preferring to participate in our study because of cultural sensitivities discussing sex and psychiatric disorders in person.

#### Sociodemographic data form

This semi-structured form included questions about sociodemographic features of the patient (i.e. age, education, occupation, marital and relationship status and number of children (if any).

#### Migraine disability assessment scale (MIDAS)

MIDAS is a tool assessing headache-related disability. Headache sufferers are asked to answer five questions scoring the number of days in the past 3 months and activity limitations due to migraine [12,13].

#### Female sexual function index (FSFI)

FSFI is a brief, self-report measure of female sexual function. It consists of 19 questions on 6-domains: desire, subjective arousal, lubrication, orgasm, satisfaction, and pain [14,15]. Each item is scored on a scale of 0 (or 1) to 5. Range for items 1, 2, 15, and 16 is 1–5, and each domain is scored with the total of the items with maximum scores of 10–20. Higher scores indicate better function, and a domain score of zero indicates no sexual activity during the past month.

#### Beck depression inventory (BDI)

BDI is a 21-item self-reporting scale developed to measure the severity of depression [16]. The Turkish version is a reliable and valid tool that is used extensively [17]. Each item can be scored from 0–3. The total score can vary from 0 to 63. BDI scores between 0-9 are considered as no depression, 10-18 as mild depression, 19-29 as moderate depression, 30-63 as severe depression. Patients that had depression according to the Beck depression scale were not excluded from the study.

#### Beck anxiety inventory (BAI)

BAI is a self-reporting scale that consists of 21 items. Each item can be scored from 0 to 3 in terms of the severity of that symptom over the past month. A high total score indicates more severe levels of anxiety; a reliable and valid Turkish form is available [18,19].

### Statistical analysis

Statistical analyses were performed using the Statistical Package for the Social Sciences (SPSS), Version 12.0. Descriptive statistics were used to summarize all measurements. For two-group comparisons, the Mann-Whitney *U* test was used. The Pearson or, if measurements were ordinal, the Spearman correlation tests were used for correlation analysis. The level of statistical significance was set as p < 0.05.

## Results

### Sociodemographics and headache-related variables

Scoiodemographics and headache-related characteristics are presented in Table 1. Briefly, mean (standard deviation; SD) age at the time of the study visit was 31.9 (6.5) years, 90% of the participants were married with a mean (SD) duration of 8.1 (6.3) years, and 50% of them had at least one child. Most patients (90%) had a diagnosis of migraine without aura with a mean (SD) duration of 7.2 (4.8) years. Mean (SD) Migraine disability assessment scale (MIDAS) score of was 19.3 (12.8); and in 40% disability was severe.

**Table 1:** Demographics and headache characteristics of the patients (n = 50)

**Demographics**		
Age	Mean (SD)	31.9 (6.5)
Working	%	70
Married	%	90
Marriage duration	Mean (SD)	8.1 (6.1)
Having at least one child	%	50
Good relationship with regular partner	%	94
**Migraine history**		
Without aura	%	90
Migraine duration (years)	Mean (SD)	7.2 (4.8)
Migraine attacks/per month	Mean (SD)	4.3 (2.7)
Mean headache intensity (0 to 10)	Mean (SD)	8.1 (1.3)
**Migraine disability assessment scale (MIDAS)**		
Mean MIDAS score	Mean (SD)	19.3 (12.8)
Minimal disability (0-5)	%	10
Mild disability (6-10)	%	22
Moderate disability (11-20)	%	28
Severe disability (>20)	%	40

### Beck depression and anxiety inventory results

Mean (SD) BDI score was 13.6 (7.9), 60% of the patients had mild, moderate or severe depression; and mean BAI score was 16.3 (0.8) (Table 2).

**Table 2:** Beck depression and anxiety inventory results (n = 50)

**Beck depression inventory (BDI)**		
Mean BDI score	Mean (SD)	13.6 (7.9)
No depression (0-10)	%	40
Mild depression (10-18)	%	30
Moderate depression (19-29)	%	28
Severe depression (30-63)	%	2
**Beck anxiety inventory (BAI)**		
Mean BAI score	Mean (SD)	16.3 (10.8)

### Sexual function

The mean (SD) FSFI score was 20.9 (5.9) out of a maximum of 36. Based on total FSFI score, 45 women had scores less than the cutoff point of 26.55, which is accepted as low sexual function [20]. The mean (SD) domain scores were as follows: desire 3.4 (1.0), arousal 4.0 (1.3), orgasm 4.2 (1.4), satisfaction 4.5 (1.5) and pain 4.9 (1.6) out of a maximum domain score of six for each.

### Correlation of sexual dysfunction with sociodemographics and headache-related findings

There was no significant correlation between FSFI total scores or any FSFI domain and age, education level, occupational status (whether the subject was actively working or not), duration of marriage, and having children or not.

Based on exclusion of patients diagnosed with moderate to severe depression according to BDI scores (n = 15), there was no significant correlation of FSFI total and sub-domain scores to MIDAS scores, migraine duration, migraine attack frequency, and migraine severity (Table 3).

**Table 3:** The correlation between female sexual function index (FSFI) total score and migraine characteristics, migraine disability assessment scale (MIDAS) score, Beck depression inventory (BDI) score and, Beck anxiety inventory (BAI) scores

	**FSFI score**													
	**Total**		**Domain scores**											
			**Desire**		**Arousal**		**Lubrication**		**Orgasm**		**Satisfaction**		**Pain**	
	r*	**p value**	r*	**p value**	r*	**p value**	r*	**p value**	r*	**p value**	r*	**p value**	r*	**p value**
**Migraine duration (years)**	−0.079	0.596	−0.237	0.109	−0.097	0.518	−0.049	0.746	0.032	0.833	−0.059	0.696	−0.094	0.531
**Migrane attack frequency**	−0.059	0.695	−0.111	0.462	−0.099	0.514	−0.089	0.556	−0.019	0.900	0.060	0.694	−0.068	0.651
**Migraine headache intensity (0 to 10)**	−0.073	0.627	−0.068	0.652	−0.034	0.819	−0.033	0.824	−0.044	0.768	0.028	0.850	−0.091	0.542
**MIDAS total score**	0.013	0.903	−0.134	0.376	0.035	0.817	−0.037	0.809	0.036	0.814	0.145	0.337	−0.042	0.784
**BDI score**	−0.486	**< 0.001**	−0.406	**< 0.001**	−0.510	**< 0.001**	−0.454	**< 0.001**	−0.470	**< 0.001**	−0.558	**< 0.001**	−0.142	0.331
**BAI score**	−0.238	0.099	−0.260	0.068	−0.234	0.102	−0.236	0.099	−0.70	0.237	−0.164	0.255	−0.203	0.158

*Correlation coefficient, bold indicates p < 0.05.

### Correlation of sexual dysfunction with depressive symptoms and anxiety

BDI scores were negatively correlated with total FSFI score (r = -0.486, P < 0.001). FSFI domain scores for desire, arousal, lubrication, orgasm and satisfaction show significant negative correlation with BDI score (r = -0.406, p = 0.004; r = -0.510, P < 0.001; r = -0.454, P = 0.001; r = -0.470, P = 0.001; r = -0.558, P < 0.001, respectively) (Table 3).

No significant correlation was detected in relation to FSFI pain domain scores and BAI scores.

### Correlation of depression scores with sociodemographics and headache-related findings

The mean BDI score was correlated with migraine attack frequency (r = 0.281, p = 0.05), but not with MIDAS total score (r = 0.144, p = 0.347), MIDAS grade (r = 0.138, p = 0.362), duration of migraine (r = -0.29, p = 0.848), severity of attacks (r = 0.162, p = 0.287) or any sociodemographic features.

## Discussion

The main aim of this study was to find whether sexual function in migraine was correlated with migraine-related disability. Our findings revealed no correlation of sexual dysfunction to the MIDAS score or MIDAS grade, although 40% of our group fell into the most severe category in MIDAS. In their study in university students with migraine, Ifergane et al. [3] reported that higher disability in MIDAS was correlated with higher health influence in sexual behavior, but not with other domains, such as satisfaction, orgasm, pain and interpersonal relationships [3], a relationship not reflected in our findings. Hence, it may be assumed that sexual dysfunction in migraine may not be the direct result of migraine related disability, and probably be moderated by other factors, at least in outpatient headache centers such as ours.

The mean (SD) FSFI score in our group was 20.9 (5.9). In a past study carried out on 1009 women in outpatient internal medicine clinics in Turkey, the mean FSFI score was reported to be 24.25 (9.50) [21]. Similarly, our group had lower mean FSFI scores compared to that reported in a population-based study and several other clinical samples [22,23] in Turkey as well as an Italian sample of primary headache sufferers treated in a tertiary university center that included postmenopausal subjects as well [5].

The reason why our group had higher rates of dysfunction than groups with other medical conditions or clinical populations with headache might either be explained by an additional negative effect of migraine on sexual function, or other clinical variables. The reasons for these low FSFI scores in our group should be further investigated in other studies in similar settings, with matched controls.

Our findings revealed no relationship of sexual function to either migraine frequency or severity. There are only a few studies examining the relationship between migraine frequency/severity and sexual function. In a recent study by Bestepe et al [4] using the Arizona Sexual Experiences Scale (ASEX), patients with migraine reported higher rates of sexual dysfunction than healthy controls but lower rates than patients with tension type headache while no relationship was detected between the affected domains of sexuality and headache frequency, severity or duration. Similarly, Maizels et al. [24] reported that the prevalence of sexual problems among migraine patients were not higher in those experiencing more (number of attacks > 2 days/week) or less frequent attacks. In their study examining the relationship between sexual desire and diagnosis of migraine, Houle et al. [6] found that subjects with migraine had a higher perceived rate of sexual desire, but could not find any correlation between headache intensity and average duration and sexual desire. There are several studies that show that levels of sexual dysfunction may be related to pain and disease severity in rheumatoid arthritis [25], fibromyalgia [26], and systemic sclerosis [27] but our results, in line with the current literature, show that sexual function is not related to clinical features of migraine.

There is a well established relationship between migraine, depression and anxiety [9,28]. Accordingly, in our study population depression was correlated with all the domains of sexual function. Despite the exclusion of patients with a previous diagnosis of depression and patients using antidepressants, the mean (SD) BDI score was 13.6 (7.9) which is lower than in some other clinical populations [29,30], but high enough to have a very significant impact on sexual function, with the identification of moderate to severe depressive symptoms in 30% of our patients. In a recent study, Nappi et al. [5] reported that depression and anxiety were very common in women treated for primary headaches (migraine and tension type) in a tertiary university center, but unlike in our results, could not find any correlation between these psychological symptoms and FSFI scores. Our results, on the other hand, depict that in patients with migraine, sexual function is significantly correlated with depression, but not with anxiety.

Depressive symptoms have been shown to be related to sexual dysfunction in the general population [31] and in large clinical samples [21]. Studies carried out in epileptic patients [32] and patients with obstructive sleep apnea [33] showed that in these medical conditions, depressive symptoms were correlated with sexual dysfunction independent of disease severity. However, duration of migraine or number of attacks per month were related to depressive symptoms but did not correlate with sexual dysfunction. Our findings seem to indicate that depression in patients with migraine is correlated with sexual function, independent of clinical features, such as migraine related disability, duration of illness, frequency or severity of attacks and anxiety.

Several studies have investigated the relationship between depressive symptoms or depression and clinical factors associated with migraine [34,35]. One study showed that depressive symptoms and insecure attachment were the most important predictors of disability in a clinical population of episodic and chronic migraine [36]. Disability was the most important determinant of the severity of depressive symptoms in primary headaches including migraine [37]. Anxiety and depression have been found to be related to MIDAS scores in outpatient settings [38], headache referral clinics [30] and in the general population [39]. Notably, depressive symptoms were significantly correlated with numbers of attacks per month in our study population, but not MIDAS total scores or migraine related disability, although numbers of migraine attacks per month were strongly associated with MIDAS scores.

The major limitation of this study is the lack of any control group and the low number of patients included, not allowing us to generalize our results. A matched control group from our outpatient department would help us explain the high rate of sexual dysfunction in our study sample. The utilization of self report instruments for the diagnosis of depression and anxiety, instead of a clinical interview, was another shortcoming.

## Conclusions

In this outpatient sample of migraineurs, sexual dysfunction seemed very common, while not related to migraine related disability, frequency of attacks, severity or anxiety. The most important factor that predicts sexual function was depression, which was also independent of disease severity and migraine related disability. In women with migraine, there may be a common factor that modulates both depression and sexual function. While future larger scale studies are needed to clarify the exact relationship, depressive and sexual problems should be properly addressed in all patients with migraine, regardless of disease severity or disability.

## Competing interests

The authors declare that they have no competing interests.

## Authors’ contributions

Category 1: (a) Conception and Design: DE; EIA; Cİ. (b) Acquisition of Data: PYD; EIA. (c) Analysis and Interpretation of Data: DE; PYD. Category 2: (a) Drafting the Article: DE. (b) Revising It for Intellectual Content: DE; PYD. Category 3: (a) Final Approval of the Completed Article: DE; EIA; PYD; Cİ. All authors read and approved the final manuscript.
